# Inkjet-Printed Bio-Based Melanin Composite Humidity Sensor for Sustainable Electronics

**DOI:** 10.1021/acsami.4c06596

**Published:** 2024-08-01

**Authors:** Peter Krebsbach, Mikel Rincón-Iglesias, Manuel Pietsch, Carmen Henel, Senentxu Lanceros-Mendez, Jun Wei Phua, Marianna Ambrico, Gerardo Hernandez-Sosa

**Affiliations:** † Light Technology Institute, Karlsruhe Institute of Technology, Engesserstr. 13, 76131 Karlsruhe, Germany; ‡ InnovationLab, Speyerer Straße 4, 69115 Heidelberg, Germany; § BCMaterials, Basque Center for Materials, Bldg. Martina Casiano, UPV/EHU Science Park Barrio Sarriena s/n, 48940 Leioa, Spain; ∥ IKERBASQUE, Basque Foundation for Science Plaza Euskadi 5, Bilbao 48009, Spain; ⊥ Insectta Pte Ltd., 77 Ayer Rajah Crescent, 139954 Singapore; # National Research Council of Italy, Institute for Plasma Science and Technology (CNR- ISTP), Via Amendola 122/D, 70126 Bari, Italy; ∇ Institute of Microstructure Technology, Karlsruhe Institute of Technology, 76344 Eggenstein-Leopoldshafen, Germany

**Keywords:** inkjet printing, melanin, humidity sensing, sustainable electronics, recycling

## Abstract

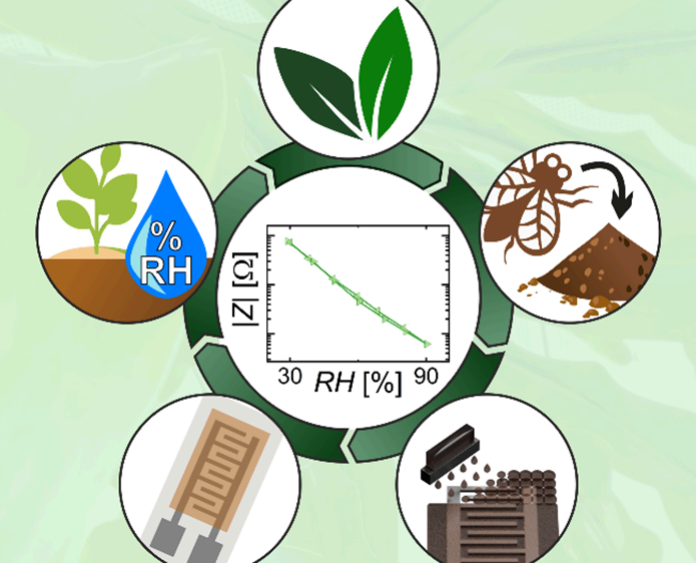

A lack of sustainability in the design of electronic components contributes to the current challenges of electronic waste and material sourcing. Common materials for electronics are prone to environmental, economic, and ethical problems in their sourcing, and at the end of their life often contribute to toxic and nonrecyclable waste. This study investigates the inkjet printing of flexible humidity sensors and includes biosourced and biodegradable materials to improve the sustainability of the process. Humidity sensors are useful tools for monitoring atmospheric conditions in various fields. Here, an aqueous dispersion of black soldier fly melanin was optimized for printing with a cosolvent and deposited onto interdigitated silver electrodes on flexible substrates. Impedance spectroscopy demonstrated that adding choline chloride increased the ion concentration and AC conductivity by more than 3 orders of magnitude, resulting in a significant improvement in sensing performance and reduced hysteresis. The devices exhibit fast detection (0.8 ± 0.5 s) and recovery times (0.8 ± 0.3 s), with a 170 ± 40-fold decrease in impedance for relative humidity changes from 30% to 90%. This factor is lowered upon prolonged exposure to high humidity in tests over 72 h during which a stable operation is reached. The low embodied energy of the sensor, achieved through material-efficient deposition and the use of waste management byproducts, enhances its sustainability. In addition, approaches for reusability and degradability are presented, rendering the sensor suitable for wearable or agricultural applications.

## 1 Introduction

The growing demand for Internet of Things (IoT) devices for use in various fields could lead to unsustainable use of resources and challenges in electronic waste (e-waste) management.^1,2^ In addition, many of these electronic components often require resource and energy-intensive fabrication and even the use of toxic materials or rare earths.^3^ In response, global efforts are underway to adopt circular economy principles and minimize environmental impact.^4,5^ However, to realize truly greener electronics, holistic approaches based on the use of sustainable raw materials and with a greater emphasis on the reusability and recyclability of products are still required. These approaches must address improvements throughout the manufacturing process, from material sourcing and device fabrication to the lifecycle of the final application.

In this context, the use of biobased low-cost byproducts from various industries as value-added products for electronic components has become a focus of intensive research in recent years.^4,6^ This approach can help both to minimize the embodied energy of the devices and increase the circularity of resources.^4^ While this selection may result in compromises in device performance compared to conventional materials, the sustainability benefits may dominate the overall impact and usefulness of the devices depending on the specific application.^4^ In particular, products designed for short-term applications must offer the possibility of sustainable end-of-life management such as harmless disposal (e.g., biodegradability), recyclability, or reusability of all its parts. These characteristics are often featured in biobased materials.^1,7−10^ The combination with energy-efficient manufacturing tools can also be beneficial in reducing the environmental impact. Printing techniques, such as inkjet printing (IJP), offer low-temperature processes and high material efficiency.^11^ Due to its digital nature, IJP deposits ink only where and when needed, minimizing the generated waste and providing scalability to industrial standards.^11,12^ However, the fabrication of printed green electronic systems from the ink formulation step to the final device is complex.^13,14^ Expanding the understanding of the complex interactions between eco-friendly functional materials, solvents, substrates, and processing steps (e.g., drop ejection, drying, annealing, etc.) is still necessary to optimize intended device characteristics.

Using the aforementioned approaches, this work focuses on the fabrication of sustainable humidity sensors. Humidity monitoring plays a critical role in various fields, from environmental monitoring for agriculture and climatology,^15−17^ healthcare and life sciences,^18,19^ to quality control for logistics, water management, and industrial processes.^20,21^ IoT sensor networks for these applications will require an enormous number of devices that can accurately determine humidity levels, with specific requirements for detection speed and sensor lifetime.^1,17,21^ Generally, three types of material classes are considered for resistive or capacitive humidity sensing: ceramics/semiconductors, organic polymers, and organic/inorganic hybrids.^17^ Literature examples with full range sensitivity for each material class can be found in Table 1. While hybrid and ceramic sensors offer advantages in mechanical strength and thermal capability,^17^ they present an increased energy consumption due to high-temperature processes and material requirements for their production.^20,22^ In comparison, organic polymer-based sensors can face disadvantages in detection speed, stability, temperature range, and hysteresis effects.^17,20,23^ However, their performance can be improved by utilizing polymer:salt electrolyte blends in the active layer by increasing the ion concentration and thereby decreasing the overall impedance.^17,24,25^ These ions experience an increased ion mobility with increasing humidity supporting the device performance.^17,26^ With that, cost-effective, flexible, and lightweight sensors can be achieved.^18,19^

**Table 1:** Overview of the Sensitivities, Detection Ranges, and Detection (*t*_90,det_) as well as Recovery Times (*t*_90, rec_) of Sensors with Different Compositions of This Work Compared to Literature Work and the EFS-10 Reference^a^

	sensitivity (30–90%)	*t*_90, det_ [s]	*t*_90, rec_ [s]	fabrication method	comment and reference
reference EFS-10	200 ± 30	4 ± 2	15 ± 3		
ChCl only	10	3 ± 3	3 ± 2	IJP	
BSF-Melanin only	2.3 ± 0.9	2.1 ± 0.7	1.4 ± 0.8	IJP	sensitivity >60% RH only
BSF-Melanin-ChCl	170 ± 40	0.8 ± 0.5	0.8 ± 0.3	IJP	
dopamine-melanin	∼30–70	6–10 s	6–9 s	drop casting	(42)
dopamine-melanin	∼700–900	0.42–0.47	0.43–0.49	drop casting	(41)
silica nanocomposite	∼15,000	110	170	coating	(22)
cellulose-KOH	∼30	6	10.8	drop casting	(23)
egg-albumin	∼1.5	>40	≥12	drop casting	capacitive; sensitivity and speeds calculated for 35% to 65%;^45^
graphene oxide	∼1400	2.7	4.6	IJP	capacitive;^46^

a The t_90_-times of this work are defined as the duration until the device reached 90% of Δ|Z| between stable wet and dry states (or vice versa). All values of sensors from this work are reported as mean and standard deviation with *n* ≥ 5 (except ChCl).

Here, we investigate a sustainable approach to fabricate inkjet-printed humidity sensors by combining the biopolymer black soldier fly (BSF)-Melanin and the salt choline chloride (ChCl) as a sensing layer. The BSF-Melanin was extracted as a byproduct from the pupal exuviae of BSF, whose larvae are used for food waste management,^27^ while ChCl is a biodegradable salt that finds application as a nutritional additive in animal feed.^28,29^ Melanins have gained interest in a variety of applications due to their abundance, biocompatibility, conductivity, and optical properties, and can be extracted or synthesized from different sources varying in amounts of dihydroxy indole and dihydroxy indole-2-carboxylic acid.^30−36^ While the exact origin and mechanism of conductivity (electrical or ionic) is still under investigation, the dependence of conductivity on the hydration of the hygroscopic melanin is well established.^37−40^ The use of melanin as a humidity sensor has been reported in the literature.^41,42^ The sensors, which utilized melanin synthesized from dopamine, responded to a broad range of relative humidity (RH) values and showed ultrafast detection/recovery times of around 0.4–0.5 s.^41^ However, they were deposited by drop casting onto vacuum-deposited gold interdigitated electrodes (IDE) and no fully inkjet-printed melanin humidity sensors are reported. To explore the potential of melanin humidity sensors using industrially relevant fabrication techniques and sustainable materials, we investigated the deposition of biosourced BSF-Melanin via IJP. ChCl was introduced as an additional ion source in the active layer to improve sensor sensitivity, resulting in absolute impedance changes of the BSF-Melanin-ChCl sensors by a factor of 170 ± 40 when measured over a wide range of RH from 30% to 90%. Furthermore, fast detection and recovery times of 0.8 ± 0.5 s and 0.8 ± 0.3 s, respectively, were achieved, which are an order of magnitude faster than those of a commercial humidity sensor. As for other polymer-based humidity sensors, the sensor exhibited instabilities upon the prolonged exposure to high humidity. Long-term tests over ∼3 days show a stable sensitivity of 27 ± 1. In addition to high performance, we demonstrated up to five times reusability of the printed metal electrodes and flexible plastic substrate with negligible performance losses. Finally, we demonstrated the sensing capabilities of the device in potential sensor applications.

## 2 Results and Discussion

**Figure 1:**
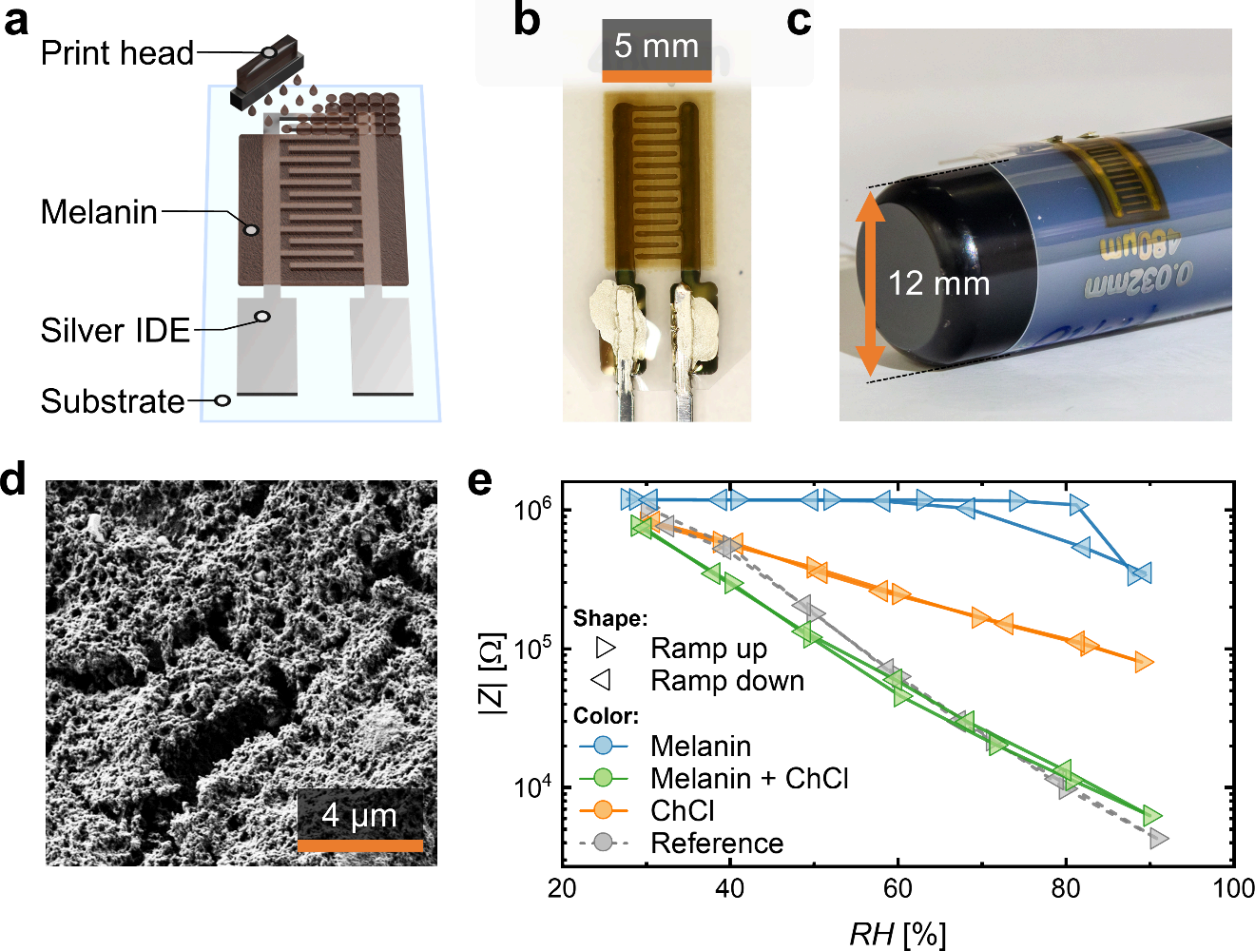
(a) Schematic representation of IJP melanin onto a silver IDE on a carrier substrate. (b) Photograph of the printed humidity sensor. (c) Photograph demonstrating the flexibility of the final, printed humidity sensor. (d) SEM image of BSF-Melanin-ChCl layer. (e) Dependency of |*Z*| on RH for different humidity sensors.

The fabrication of the BSF-Melanin humidity sensor involved the printing of two successive layers. Figure 1 schematically shows the fabrication as well as morphological and functional characterization of the inkjet-printed BSF-Melanin humidity sensor. First, an Ag IDE was deposited onto a polyethylene naphthalate (PEN) substrate. Subsequently, a layer of an optimized ink based on BSF-Melanin was inkjet-printed on top, as depicted in Figures 1a and 1b, using an optimized water-based eco-friendly ink formulation comprising of ChCl, BSF-Melanin and the cosolvent triethylene glycol monomethyl ether (TGME). The latter was used as a biocompatible, and biodegradable ink additive with low toxicity that allows better drying and printability due to its higher boiling point (∼250 °C) as well as suitable surface tension (γ) and (dynamic) viscosity (η).^43,44^ ChCl was used to increase the ion concentration and thus the AC conductivity σ_AC_. The humidity sensing layer (i.e., BSF-Melanin plus ChCl) was homogeneously printed over several mm while the devices exhibited a large degree of mechanical flexibility (Figure 1c). Other salts such as NaCl and CaCl_2_ led to the formation of less homogeneous films (Figure S1). Next to the benefits of freedom of pattern design, IJP could control the layer thickness and drying speed, which helped to reduce the cracking of the layer. For comparison, Figure S2 shows a drop cast film of a BSF-Melanin layer from high-concentration ink that cracked and detached from the substrate during the drying process. The micromorphology of the films was investigated by Scanning Electron Microscopy (SEM). Figure 1d shows that the composite formed a porous film providing a large surface area for the sensor to interact with the surrounding environment. The performance of the BSF-Melanin-ChCl humidity sensor is presented in Figure 1d where we compare it to the performance of the single component sensors as well as a commercially available electrolytic reference sensor (EFS-10). The characterization of the sensors was carried out by recording their absolute impedance |*Z*| (at 1 kHz) inside a climate chamber held at 25 °C while cycling RH from 30% to 90% in steps of 10% to allow for the investigation of hysteresis effects.

The fabricated sensor exhibited an exponential decay in impedance from 570 kΩ to 3 kΩ with negligible hysteresis at 0.6 V, closely overlapping with the response of the commercial device and outperforming the pure BSF-Melanin and pure ChCl sensors. To quantify the difference among devices, we define the sensitivity value as the ratio from the maximum and minimum |*Z*| at 30% and 90% RH. Their average value and their humidity detection range are presented in Table 1. It is observed that our BSF-Melanin-ChCl sensor reached a sensitivity value of 170 ± 40 which is comparable to that of the EFS-10 reference (200 ± 30). As Table 1 presents, ceramic or silica nanocomposite and graphene oxide sensors show significantly higher sensitivities (∼15000 and ∼1400, respectively) than the BSF-Melanin-ChCl sensor, while other biodegradable or melanin-based humidity sensors reach similar orders of magnitude for changes from 30–90% RH.^22,23,41,42,45,46^ Most of these humidity sensors are not fabricated using scalable printing techniques with drop casting being the most common method. The inkjet-printed BSF-Melanin-ChCl demonstrates a combination of sensitivity with fast detection (see below) and controlled deposition. The BSF-Melanin single-component devices reached substantially lower sensitivities compared to the BSF-Melanin-ChCl sensors by an order of magnitude. In the case of pure BSF-Melanin, the overall sensitivity reached 2.3 ± 0.9 with significant changes occurring for >60% RH. In this range, |*Z*| decreased from ∼1.2 MΩ to 335 kΩ and showed a hysteresis that would further impair the precise reading of a sensor. Pure ChCl could not be processed into a reproducible, mechanically stable layer. Only one pure ChCl device showed a response in the climate chamber so no statistics could be collected. Therefore, the combination of BSF-Melanin and ChCl was not only beneficial to achieve a higher sensitivity but also to improve the structural resistance of the material and thereby fabrication yield.

**Figure 2:**
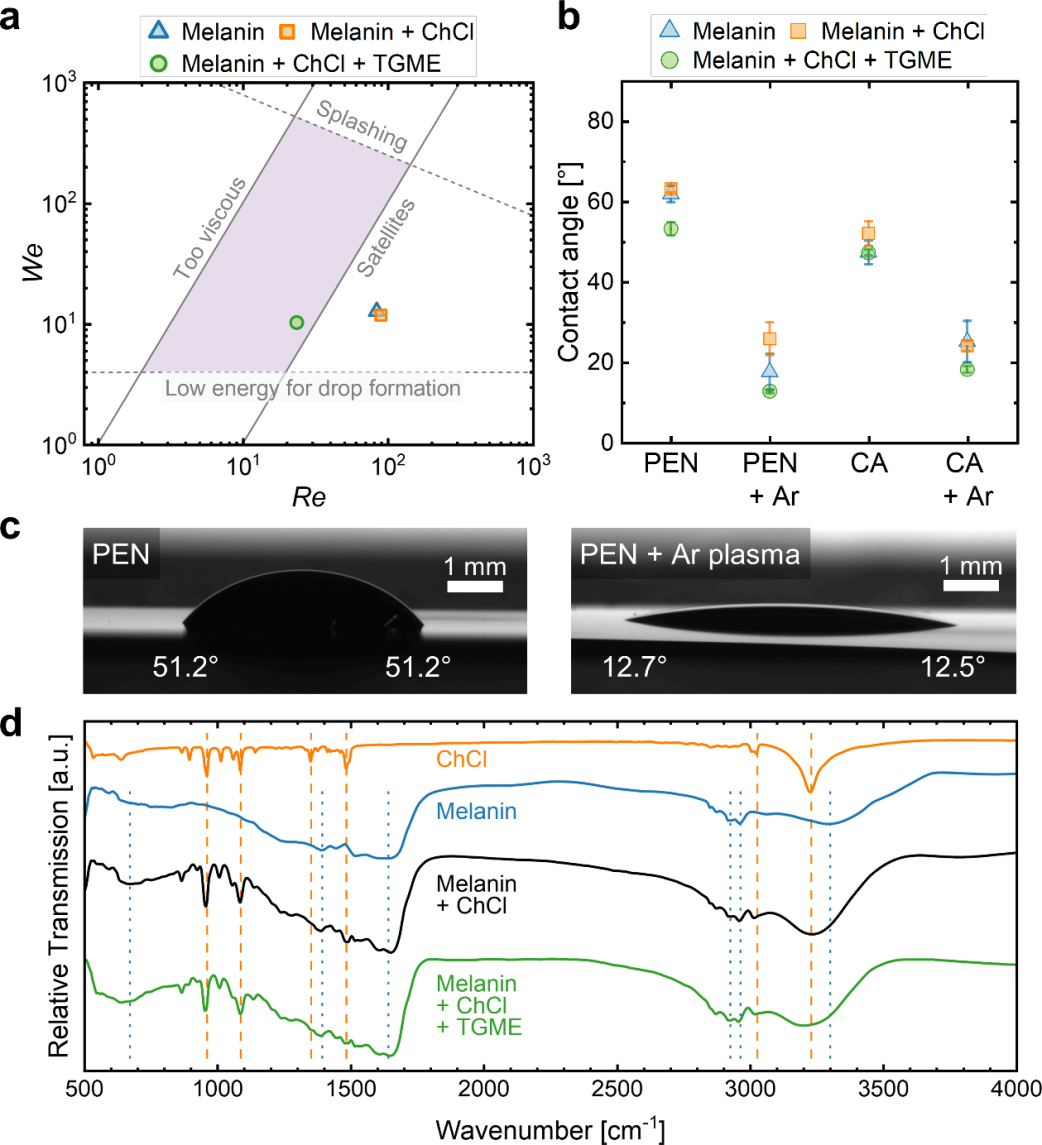
(a) Representation of inkjet printability with the optimal range marked in purple. The Re and We of three different combinations of melanin, ChCl, and TGME are indicated in the plot. (b) Contact angle (mean values with standard deviation) of the inks on PEN and cellulose diacetate (CA) with and without prior Ar-plasma treatment (1 min). (c) Photographs of contact angle measurements on PEN for melanin-ChCl-TGME ink without and with Ar-plasma. (d) FTIR transmission spectra of material combinations of melanin, ChCl, and TGME. Peaks assigned to ChCl and BSF-Melanin are indicated with orange dotted and blue dashed lines, respectively.

Inkjet printing from water-based solutions is typically challenging because of high γ, low *η,* and low boiling points which commonly result in nozzle clogging and nonideal film drying (e.g., coffee ring formation).^14,47,48^ Adding a suitable cosolvent is therefore often used to improve the printability of the ink by tuning η, γ, and/or contact angle on the substrate.^47,49^ A graphical representation of the printability of an ink formulation is shown in Figure 2a where its Weber number (*We* = *v*^2^·ρ·*a*·γ^–1^, *v*: drop velocity, ρ: density, *a*: nozzle diameter) is plotted against its Reynolds number (*Re* = *v*·ρ·*a*·η^–1^).^50^ Potentially printable inks should fall into the colored box whose top and bottom boundaries are set by the onset of splashing at *We*^1/2^·*Re*^–1/4^ > 50 and minimum energy for drop formation at *We* > 4.^50,51^ The right and left boundaries are given by the Ohnesorge number (*Oh* = *We*^1/2^·*Re*^–1^). A typical range for stable droplet formation in IJP is defined as 10 > 1/*Oh* > 1.^52^ Using values for *a* = 16.5 μm and *v* = 5 m s^–1^ and the measured properties of the inks (Table 2), both water-based formulations (i.e., pristine BSF-Melanin and BSF-Melanin-ChCl) fell outside of the optimal area. During our printing experiments, we could not achieve stable drop formation with either of these inks. The addition of the high boiling point, nonhazardous, and biodegradable solvent TGME in a 1:2 ratio to water increased η and decreased γ of the ink moving its *Re* and *We* numbers into the optimal area (Figure 2a) and allowing for successful drop formation.^44^

**Table 2:** Overview of Ink Properties^a^

	η [mPa s]	γ [mN m^–1^]	ρ [kg m^–3^]
BSF-Melanin	1.28 ± 0.09	43 ± 4	1007 ± 2
BSF-Melanin-ChCl	1.2 ± 0.2	46 ± 3	1010 ± 2
BSF-Melanin-ChCl with TGME	3.6 ± 0.2	41 ± 1	1030 ± 10

a All inks are water-based.

When ejected drops reach the substrate, a suitable interaction with the substrate is needed to yield a low contact angle that allows for drop coalescence and homogeneous film formation through controlled drying. The formulated inks exhibited contact angles on PEN between 50° and 65°. We achieved lower contact angles <25° by a 1 min Ar-plasma treatment of the substrate, as shown in Figures 2b and 2c. The successfully printed layers in Figure 1b were achieved by additionally raising the temperature of the substrate table to 40 °C during printing. The elevated temperature helped to hold the ink in place during printing and reduced shrinkage effects after water evaporation.

To exclude chemical interaction between the counterparts of the sensing layer deposited from pure water or water-TGME, Fourier-Transform Infrared (FTIR) spectroscopy measurements were conducted (Figure 2d). The spectrum of BSF-Melanin deposited from water solution showed characteristic absorption peaks located at 3300 cm^–1^ (NH- and OH-stretching), 2925 cm^–1^ (CH-stretching), 1640 cm^–1^ (C = C and C = N bending of aromatic ring and aromatic system, C = O of carboxylic group), 1392 cm^–1^ (aliphatic C–H), and 670 cm^–1^ (alkene C–H). The position of the absorption peaks are indicated in Figure 2d as blue dashed lines and agree with values reported for melanins extracted or synthesized from other sources.^53−56^ Pure ChCl showed its characteristic peaks in the range from 850 to 1500 cm^–1^ (C–N, C–H and C–O stretching) and 2650 to 3600 cm^–1^ (-H and C–H stretching) with the most prominent peaks at 960, 1090, 1350, 1480, 3030, and 3230 cm^–1^ (orange dotted lines).^57−59^

The dominant peaks from both spectra could be identified in the composite film deposited from a purely water-based solution, where no changes in their positions or relative absorption strength were observed. Similarly, the spectrum of the layer deposited from a water-TGME-based formulation showed neither peak shifts nor the appearance of new peaks. No dominant contributions of TGME to the absorption of the composite were observed. According to the literature, the strongest absorption from TGME would be detectable around 1108 and 2877 cm^–1^.^60,61^ Thus, we concluded that the addition of TGME had no structural effect on the composite and thereby no impact on sensing performance. It solely served to enable the IJP process. Also, in UV–vis spectra, no distinct changes were observed (Figure S3). It can be noted, that this addition was also successful for the printing of BSF-Melanin-water-TGME dispersion without ChCl (Figure S4).

**Figure 3:**
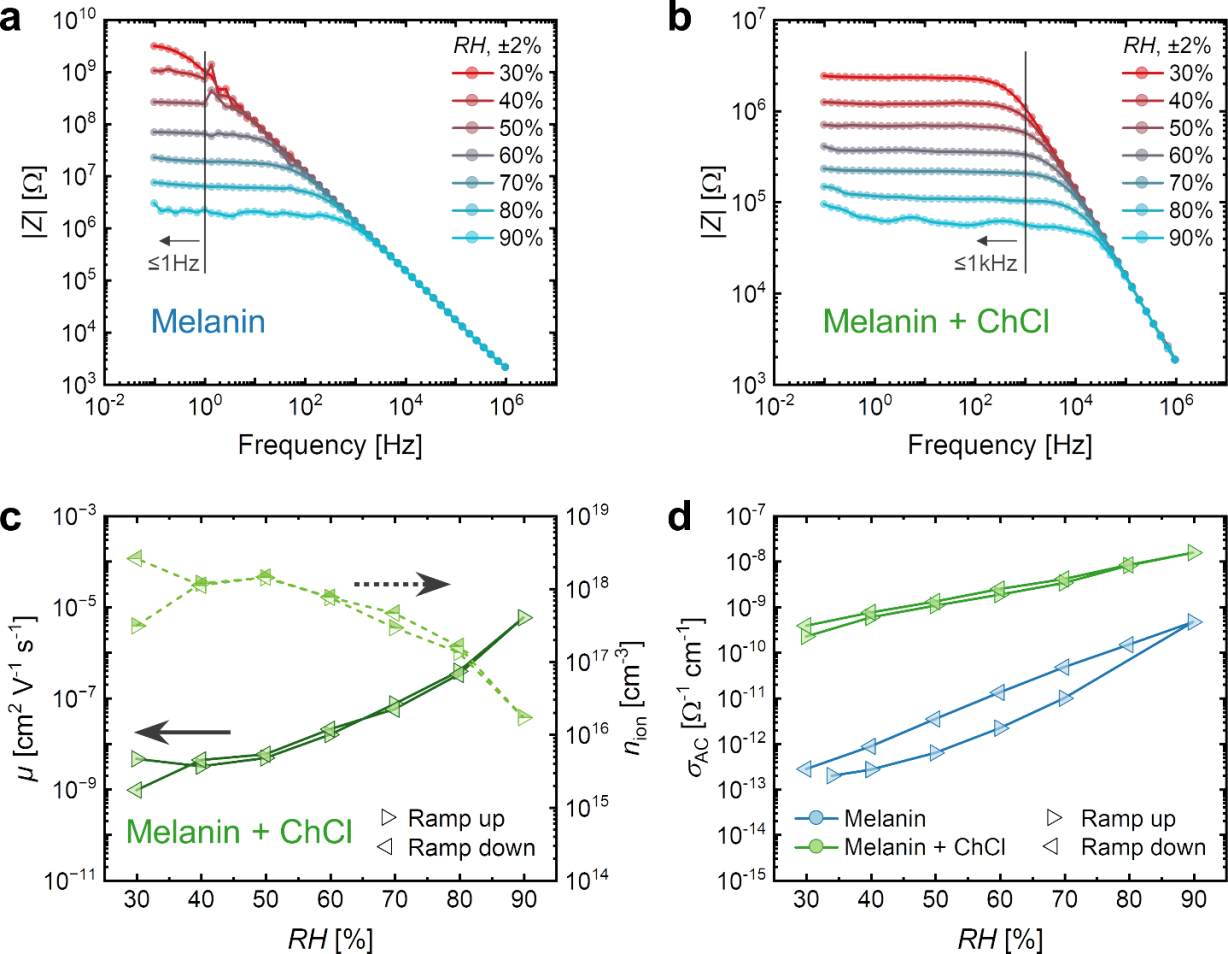
(a) The frequency response of a pure melanin sensor at different humidities. RH was ramped from 30% to 90%. The vertical line indicates frequencies below which a full detection range is possible. (b) The frequency response of a melanin-ChCl sensor. (c) Calculated μ (dark solid line) and *n*_ion_ (light dashed line) of the BSF-Melanin-ChCl sensor plotted against RH. The data is shown for increasing (ramp up) and decreasing (ramp down) RH. (d) Calculated σ_AC_ of the sensing layers plotted against RH.

The influence of ChCl on the sensitivity of the sensors was investigated using electrical impedance spectroscopy (EIS) measurements. Figures 3a and 3b compare the frequency dependence of |*Z*| of inkjet-printed sensors with pure BSF-Melanin and BSF-Melanin-ChCl sensing layers as a function of RH at 0.3 V. Both devices were deposited from water-TGME-based ink formulations. The Bode plots showed the typical |*Z*| frequency response of an RC circuit with a plateau at low frequencies and a corresponding decrease at higher values. For both samples, the maximum value of |*Z*| decreased with an increase in RH, suggesting an increase in σ_AC_. For the pure BSF-Melanin sensors, significant changes in all measured RH values were observed at frequencies <1 Hz. Above this frequency, we detected a reduction in dynamic range until all curves overlapped above ∼1 kHz. This explains the smaller detection range observed for this sample (see Figure 1e) by the LCR meter, which has an operational frequency of 1 kHz. The addition of ChCl (Figure 3b) led to a reduction of |*Z*| down to values between 10^4^ and 10^6^ Ω. The composite device exhibited a bandwidth that was 3 orders of magnitude larger than the pristine BSF-Melanin device, which enabled full-range measurements with high sensitivity at operational frequencies up to 1 kHz.

The differences in performance after the addition of ChCl could be explained by the variation in carrier mobility μ, σ_AC_, and ion concentration *n*_ion_. These values can be estimated by analyzing the EIS data to calculate the permittivity and σ_AC_ considering the geometry of the device (for details, see Note S1 in SI and Figure S5). Figure 3c shows the calculated values for μ and *n*_ion_ of the BSF-Melanin-ChCl sensor, while data for a pristine BSF-Melanin sensor is shown in Figure S6. Both sensors exhibited a strong increase in μ with water uptake as RH increased. Despite its lower μ compared to the pristine BSF-Melanin, the observed |*Z*| reduction of the BSF-Melanin-ChCl sensors could be explained by a 3 to 4 orders of magnitude increase in *n*_ion_ up to ∼10^18^ cm^–3^ (Figure 3c, right axis). This increase corresponded to the observed rise in the calculated σ_AC_ (Figure 3d) when compared to the pristine sensor. Again, a reduced hysteresis was observed for the composite sensor with good agreement between the data points of rising and falling RH. Overall, through the addition of ChCl, we were able to increase σ_AC_ as well as the cutoff frequency of the sensor and thus obtained faster devices with a wider operational window.

**Figure 4:**
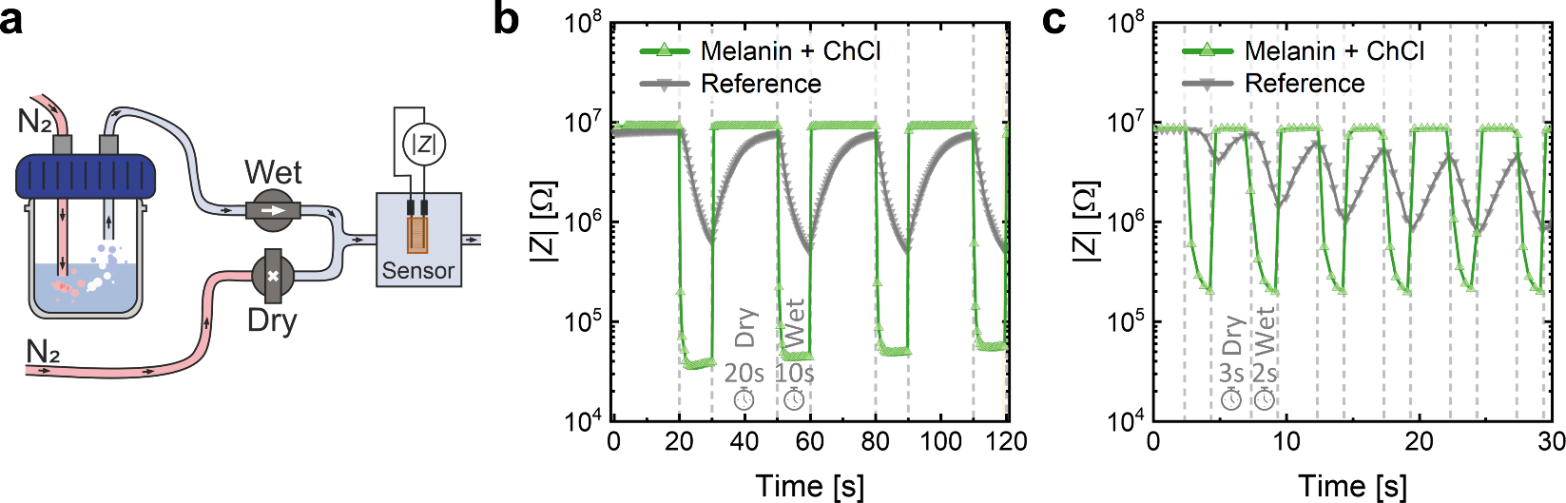
(a) Schematic representation of the dynamic measurement setup. Nitrogen gas was directed purely (dry) or via a water bottle (wet) to a sample box. Valves were used to switch between the gas streams. |*Z*| of the sensor inside the box was monitored while alternating dry and wet conditions. (b) Monitored |*Z*| over time for the melanin-ChCl and reference sensor. 20 s of dry and 10 s of wet nitrogen were cycled. **c** For faster cycling, 3 s of dry and 2 s of wet nitrogen streams were used.

The dynamic response of the sensors to rapid changes in RH was investigated by exposing the sensor to wet or dry N_2_ gas in a customized setup as schematically represented in Figure 4a. |*Z*| of the BSF-Melanin-ChCl sensor and the EFS-10 reference were monitored as a function of time and alternating dry and wet N_2_ streams as shown in Figures 4b and 4c. It is observed that the printed BSF-Melanin-ChCl sensor outperformed the commercial device. To quantify this, we defined a *t*_90_ value representing the time the sensor needs to reach 90% of Δ|*Z*| between stable wet and dry states. The printed BSF-Melanin-ChCl sensors rapidly adapted to changes in RH with *t*_90, det_ = 0.8 ± 0.5 s when changing from dry to wet N_2,_ and *t*_90, rec_ = 0.8 ± 0.3 s for the opposite change. Assuming negligible temperature difference for the laboratory and the climate chamber (∼2–3 °C) and using an exponential fit to the calibration of the used sensor in the climate chamber (Figure S7), the wet and dry states were approximated to 84 ± 2% and 3 ± 1% RH, respectively. It could be observed that the reference sensor did not reach a stable value within the 10s exposure to wet-N_2_. In the datasheet, a *t*_90_ of <120 s is provided, while in our measurements a *t*_90, det_ of 4 ± 2 s and *t*_90, rec_ of 15 ± 3 s were determined (see Figure S8).

Faster cycling times (Figure 4c) showed that neither sensor fully reached a stable detection level within 2 s of wet N_2_ stream. Yet, for the BSF-Melanin-ChCl sensor a quick and full recovery was observed in the dry period while the reference sensor could not detect or regenerate, leading to continuously decreasing |*Z*|. We did not perform measurements at faster rates since the utilized LCR meter had a maximum sampling frequency of 2 Hz. While pure BSF-Melanin films exhibited a *t*_90, det_ of 2.1 ± 0.7 s and a *t*_90, rec_ of 1.4 ± 0.8 s, their sensitivity range was significantly lower as described above (Figure S9). Sensors with pure ChCl gave unreliable and strongly varying results (Figure S10). The average detection and recovery times for all mentioned sensors are summarized in Table 1 and show faster operation than most cited literature.

The fast detection and recovery of the BSF-Melanin-ChCl sensor enable its potential use in dynamic RH sensing applications. The biocompatibility of the BSF-Melanin, its potential biodegradability, the wide range of detectable RH at high speed, and the mechanical flexibility of the sensor make it ideal for wearables or agricultural applications. Furthermore, the benefits of processing samples by IJP onto a flexible substrate, such as freedom of design and the ability of customization, lightweight, high throughput, and low cost, facilitate the transfer to industrial processes.^11,12,14^ A selection of potential applications was investigated.

**Figure 5:**
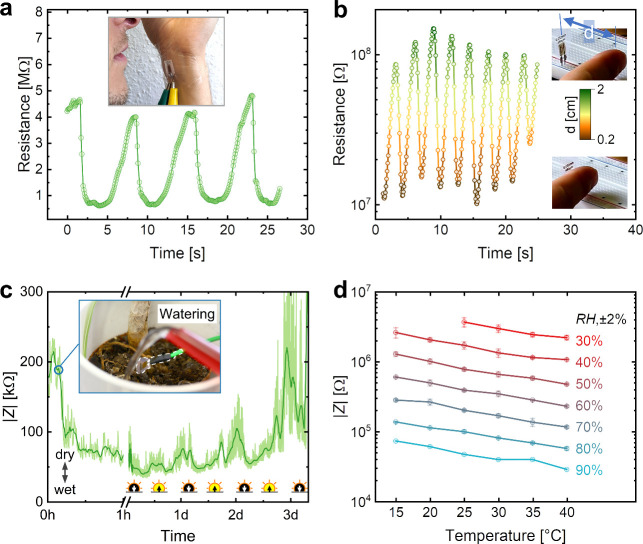
(a) Humidity sensor used as a breath sensor. The sensor was attached to the arm as can be seen in the inset and deep, slow breathing was recorded. (b) Humidity sensor used as a finger proximity sensor. A finger was periodically moved close to the sensor (0.2 cm) and away (2 cm). (c) Humidity sensor used for soil monitoring. The initially dry soil of a plant was monitored during the dry state, the watering, and the following days. Shown is the recorded data in light green and the moving average in dark green. Sun symbols indicate the times of sunrise and sunset. Note the change in scale for the time axis. (d) Temperature dependency of |*Z*| for different RH values. Values are means with their standard deviation.

Figure 5a presents a printed BSF-Melanin-ChCl breath sensor. The sensor PEN substrate was attached to the subject’s arm with medical tape. The sensor was held in front of the mouth and nose at a distance of ∼5 cm. A calm, slow, deep breath at rest was recorded via the DC change in the electrical resistance of the humidity sensor. Starting at ∼4.5 MΩ, the resistance drops <1 MΩ during the humid phase of exhalation. During inhalation, the resistance increases back to values above 4 MΩ. From the detection on-set, the respiratory rate could be determined to be ∼8.3 min^–1^. The inkjet printability of the humidity sensors allows for greater design flexibility, enabling them to be incorporated into face masks or tubes for monitoring the breathing of sick or intubated individuals. Another application for the humidity sensor can be proximity sensing. A humidity cloud extending a few millimeters typically envelops the human skin, making it possible to use a humidity sensor as a proximity sensor.^62^ For this purpose, we recorded the resistance of the sensor during the periodic movement of the subject’s finger from a distance of 2 cm to ∼0.2 cm (Figure 5b) as the sensor’s reading showed the highest sensitivity in this range. Variations between cycles could be due to slight variations in finger movement as well as the influences of the subject’s breathing and body temperature on the sensing environment. Given the large variation in skin type and skin moisture for different individuals or their physical states, a proximity sensor for absolute readings seems difficult to calibrate. Instead, the humidity sensor could be used for human-machine interfaces, security mechanisms, or electronic skins and adapted to necessities in shape or flexibility.

Next, our sensor was used to monitor soil humidity changes in an indoor plant (*Dracaena marginata*). The sensor was suspended closely above the soil while its |*Z*| was continuously recorded. Figure 5c shows the collected impedance data (light green) and its moving average (dark green; moving average over 2 min in first 1 h, then over 50 min). After 10 min of recording the initial dry state, the pot was watered and the sensor impedance level was monitored over 3 days. A clear decrease in |*Z*| of more than 100 kΩ could be observed after watering the plant. |*Z*| continued to decrease over the next 6 h, indicating an increase in humidity on the top of the soil. The trend of |*Z*| then reversed and increased continuously in the next days, indicating slow drying of the soil and reduced humidity at the sensor. However, small peaks of about half a day were observed. Possible variations in the office environment might have affected the recording. In addition, it is known that plants undergo diurnal patterns that could influence the humidity of the soil through water uptake or release by the plant. These changes occur at the same frequency as the oscillations detected by our sensor.^63,64^ The exact origin of these peaks was not investigated further. Nevertheless, it was demonstrated that the sensor could be used as a tool for smart farming and precision agriculture to monitor the humidity levels of crops and other plants. Future work could investigate environmental aspects (rain, temperature, UV exposure, ...) in these scenarios and their influence on device performance and long-term stability.

For many applications, the temperature dependency or stability of the sensor and its reading is important. The |*Z*| change of the sensor as a function of temperature and RH can be found in Figure 5d. It can be observed that the printed BSF-Melanin-ChCl sensors showed a uniform dependency of |*Z*| on temperature for all RH values. A calibration to temperature changes through parallel temperature readings could therefore be possible.

**Figure 6:**
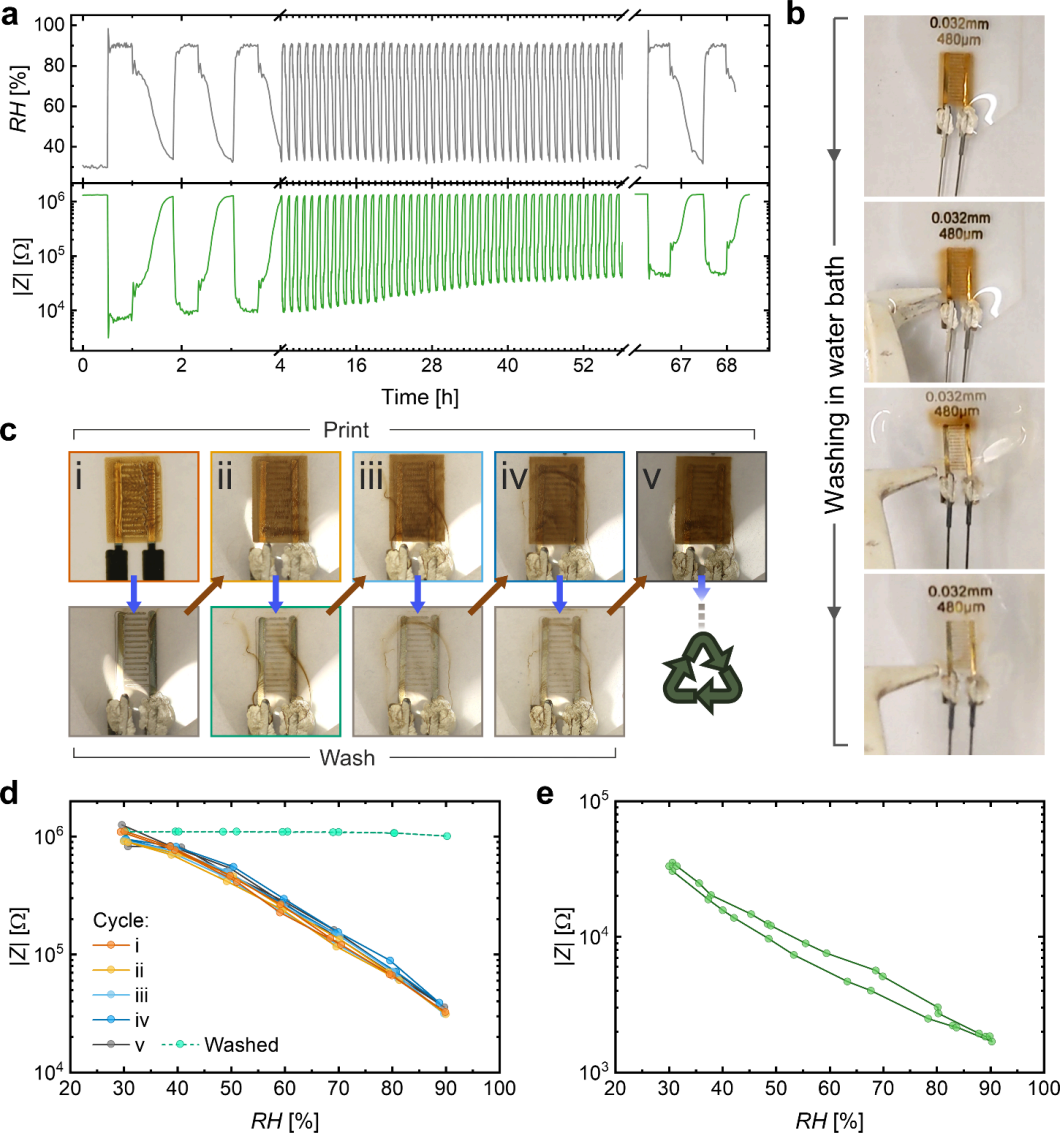
(a) Long-term stability of a humidity sensor evaluated under hourly cycling RH conditions. Top panel: Data from the built-in sensor of the climate chamber. Bottom panel: Recording of |*Z*| of the BSF-Melanin-ChCl sensor. (b) From top to bottom: Removal of the sensing layer. The sensor was immersed in a water bath (top) and taken out (center images) so that a cleaned IDE on PEN was obtained (bottom). (c) Cycling experiment: printing sensors, washing the active layer, and reusing the same substrate and IDE for subsequent printing in five cycles. (d) Characterization of the sensor shown in panel c in the climate chamber after different printing cycles and after the washing step. (e) Humidity sensing of a potentially biodegradable BSF-Melanin-ChCl sensor. The active layer is printed onto printed gold electrodes on a CA substrate.

The long-term stability of the BSF-Melanin-ChCl sensors was investigated through a stress test over ∼67 h (Figure 6a). For this, the climate chamber was programmed to alternatingly hold the extreme values of 30% or 90% RH for ∼30–40 min each. The upper panel of Figure 6a shows the recorded data from the chamber’s built-in sensor. It can be seen that humidifying the chamber takes a much shorter time than drying to 30% RH. The bottom panel of Figure 6a shows the recording of |*Z*| of a printed BSF-Melanin-ChCl sensor. The sensor closely reproduced the dynamics of the climate chamber. However, the sensitivity of the sensor decreased from 182 ± 15 in the first cycle to 29 ± 1 in the last due to the harsh exposure to 90% RH. The strongest changes in sensitivity were observed between the first cycles while they got weaker after around 40 h. Harsher conditions (no full recovery to 30% RH) led to a faster settlement of the sensitivity and resulted in the comparable final sensitivity of 26.9 ± 0.8 that did not significantly change within additional 3 days of continuous exposure to 90% RH (27 ± 1, Figure S11). The instability of the sensors was also reflected in morphological changes of the layer after measurements as could be observed under the microscope (Figure S12). The reason for the changes was most likely due to the persistent water solubility of the deposited layer and reflects a common issue for polymeric humidity sensors.^17^

Yet, the biosourced materials, ease of fabrication with low embodied energy, and biocompatibility open the door for the aforementioned, suitable applications with short life cycles in a circular economy. To this end, two concepts of reusability of sensor parts and biodegradability are discussed below, which could increase the sustainability of the use of the devices. Reusability reduces the burden on precious metals for electrodes and substrate materials even for recyclable plastics (e.g., PEN). To demonstrate the direct reusability of sensor parts, a sensor was printed and characterized as described above, placed in a water bath for approximately 1 min (top image Figure 6b), and then retrieved (bottom three images Figure 6b), after the BSF-Melanin-ChCl layer had visibly dissolved. Nevertheless, the cleaned substrate with IDE could be reused for IJP of the BSF-Melanin-ChCl layer as demonstrated in Figure 6c. Thus, almost unchanged sensor performance was achieved for the second-generation sensor, saving substrate and electrode material. Without the sensing layer, the remaining electrodes showed no response to changes in RH compared to the initial sensor, as shown in Figure 6c. The entire experiment was repeated five times as a proof of concept with negligible variations in the |*Z*|-RH dependence. Finally, defects in the silver electrodes or substrate could eventually end the reusability of the sensor. In such a case, the sensor could eventually be washed, the thin electrode material recovered and the PEN substrate recycled.^65,66^

Finally, a potentially fully biodegradable inkjet-printed sensor was demonstrated using printed gold as the electrode and cellulose diacetate (CA) as the biodegradable substrate material. The compatibility of the substrate with the inkjet process of BSF-Melanin was demonstrated in Figure 2, where the contact angle of CA was determined to be <50° without and <20° with prior plasma treatment. The sensor showed a decrease in |*Z*| with higher RH following an exponential decay and a sensitivity of 19 ± 1 (Figure 6e). The overall lower |*Z*| values compared to the sensor on PEN and Ag IDEs could be explained by the different electrode dimensions and material conductivity (see Note S2 in Supporting Information for details on fabrication differences). Such a sensor could be used for agricultural monitoring and could potentially be fully degradable in the soil, reducing the environmental impact. Further improvements in biodegradability and reduced environmental impact could be achieved with carbon-based electrodes. In addition, future work could also concentrate on chemical modifications of the active layer to prolong, enhance, or trigger its controlled degradation via specific stimuli (e.g., temperature, UV illumination). For the final choice of biodegradability and reusability or the use of both, a life cycle assessment for the specific application can be performed and the most appropriate technique applied for increased sustainability.

## 3 Conclusion

In conclusion, a sustainable approach for inkjet-printed, biobased BSF-Melanin-ChCl humidity sensor was demonstrated. The addition of ChCl enabled sensors with a high sensitivity of 170 ± 40 in a wide range of RH (i.e., 30% to 90%). Compared to a commercial reference sensor, the printed BSF-Melanin-ChCl sensors showed about an order of magnitude faster detection and recovery times down to 0.8 ± 0.5 s and 0.8 ± 0.3 s, respectively. Long-term measurements in high humidity demonstrate a settlement to a stable sensitivity of 27 ± 1. Potentially, cross-linking of the polymer could improve the device in further studies, where the biodegradability of the cross-linked material and electrode should be considered. To increase sustainability by reducing material consumption, we have shown that the polymer substrate and metal electrodes of the sensor can be reused at least five times. Additionally, the sensor could be processed on a biodegradable substrate and demonstrated potential use for applications in agriculture, healthcare, or proximity sensing. Future work on inkjet-printed melanin sensors could also investigate their response and sensitivity to other analytes. By using BSF-Melanin as a high-performance sensing material, we showed that a low-cost byproduct from the biowaste management chain can be transformed into a value-added material for electronics. Therefore, our approach represents a viable route to address sustainability and e-waste issues while still meeting the requirements for IoT sensors.

## 4 Experimental Section

### Materials

BSF-Melanin was kindly provided by Insectta Pte Ltd. The BSF-Melanin powder is extracted from BSF pupal exuviae–a byproduct of BSF farming–yielding dispersible nanoparticles.^27^ TGME, silver nanoparticular ink (Silverjet), and choline chloride (ChCl, 99%) were purchased from Merck. As substrates, PEN (Teonex Q5100, Pütz GmbH+Co Folien KG) or CA (150 μm, Rachow-Kunststofffolien GmbH) were used. Gold ink (DryCure Au-J, 1010B) was purchased from C-ink.

### Ink formulation

First, pure BSF-Melanin films were prepared by dissolving BSF-Melanin powder in deionized water at a concentration of 4 wt %. For BSF-Melanin-ChCl films, 3 wt % of ChCl (relative to water) were added. For optimized inks, 50 vol % TGME (relative to water) was added. After mixing all components, all inks were vortexed for 15 s, filtered (polyvinylidene fluoride filters, 0.45 μm pore size, 13 mm diameter; Whatman Puradisc), and degassed in an ultrasonic bath for 15 min before further use. For high loadings of BSF-Melanin and ChCl, more than one syringe filter could be required to filter all the ink.

### Device Fabrication

The substrates were treated with 20 s of Ar-plasma (Nano and Pico, Diener electronic GmbH & Co. KG) to ensure wettability. IDEs were inkjet-printed with filtered Ag nanoparticle inks on PEN using a PiXDRO LP50 (Süss MicroTec SE) inkjet printer with a Dimatix Materials Cartridge Samba printhead. The layers were cured at 120 °C for 10 min. The IDEs had a line spacing of 282 ± 5 μm, a finger length of 1.84 ± 0.05 mm, and a finger width of 228 ± 3 μm. The thickness of the silver IDEs was 58 ± 7 nm when printed at 800 dpi. The print resolution and finger spacing of the IDE have been selected due to conductivity and performance optimization (see Figure S13).

Inkjet-printed deposition of rectangular BSF-Melanin layers was done with DMC Samba printheads (Fujifilm) heated to 35 °C at a resolution of 4000 dpi and onto a heated substrate at 40 °C. Next, the wet layer was dried on a hot plate at 120 °C for 5 min resulting in ∼1.5 ± 0.3 μm thickness.

Crimping contacts (CrimpFlex, Nicomatic SA) were used to establish electrical connections to the flexible sensors. To ensure the electrical connection, a conductive silver paint (Ferro GmbH) was manually applied to the printed contact pad-crimp contact interface.

For recycling experiments, BSF-Melanin-ChCl sensors were printed and measured in the climate chamber as described below. After the characterization, the sensors were washed via immersion in a water bath for ∼1 min. Afterward, the cleaned IDE on PEN was dried and then used without further modification for the second and subsequent cycles of printing, characterizing, and washing.

### Ink Characterization

The viscosity of inks was determined with an m-VROC Viscometer (RheoSense). The contact angle and surface tension of inks and the free surface energy of substrates were measured with a KRÜSS DSA 100 drop shape analyzing system.

### Film Characterization

For film characterization, a stylus profilometer (Dektak 150, Bruker) was used to determine the layer thickness. Micrographs were taken using an optical microscope (Nikon Eclipse 80i) and a SEM (Zeiss Auriga System) at 1 kV in high vacuum. FTIR measurements were taken under vacuum with a Bruker Vertex 80v using a nitrogen-cooled mercury cadmium telluride detector and an average of over 200 scans at a resolution of 4 cm^–1^. Layers for FTIR were deposited via drop casting on silicon substrates. Transmission results are shown relative to silicon.

### Device Characterization

The electrical characterization of the humidity sensors was performed in a climate chamber (MKF115, Binder GmbH) that allows synchronous regulation of temperature and RH. The temperature was kept constant at 25 °C and RH changed in steps of 10%. The duration of measurement (especially EIS) and the chamber’s stability at constant RH influence the real RH and thereby values in this work are given within ±2% (especially for >85% RH). The sensors in the chamber were connected via cables to the kelvin test clips of a hand-held LCR meter (ST2822E, Sourcetronic GmbH) to measure absolute impedance at 1 kHz and 0.6 V_rms_ once the climate chamber stabilized. For each RH, five measurements were taken of which their average value and standard deviation were used for the evaluation. Electrochemical impedance spectroscopy (Autolab, Metrohm) was performed with sensors in the same climate chamber for frequencies between 0.1 Hz and 1 MHz for a 0.3 V_rms_ sine wave. For long-term measurements, the climate chamber was programmed to cycle between 30% and 90% RH for 30–40 min each. Measurements were compared to a commercially available, electro-analytic polymer sensor (EFS-10, B+B Thermo-Technik GmbH).

Dynamic measurements to evaluate the detection and recovery speed of sensors were done with a custom-built setup that allows for dry or wet nitrogen gas flows toward a small sample chamber of approximately 6 × 6 × 6 cm^3^ (see Figure 4a). Gas valves were used to manually change between the gas streams. The LCR meter records absolute impedance values at 1 kHz via a Python script (sampling frequency ∼2 Hz). Resistance measurements were done with a Keithley 2400 SMU. For application tests including a subject, written consent was obtained prior to testing. The sensor was attached to the arm using medical tape (3M Tegaderm Roll) with no direct contact of the sensor to the skin.

## Supporting Information Available

Microscope images and photographs of layers and devices, UV–vis spectra, details on data analysis and additional data for EIS, additional data for dynamic characterization and long-term experimentation, and notes on fabrication differences (PDF)
