# Supplementary material for: Inkjet-Printed Bio-Based Melanin Composite Humidity Sensor for Sustainable Electronics
Source: ACS Appl Mater Interfaces. 2024 Aug 1;16(32):42555–65. doi: 10.1021/acsami.4c06596 (PMC11332400; doi:10.1021/acsami.4c06596)
Supplement: Supplementary file 1 — am4c06596_si_001.pdf [file am4c06596_si_001.pdf]

# SUPPORTING INFORMATION

## Inkjet-Printed Bio-Based Melanin Composite Humidity Sensor for Sustainable Electronics

*Peter Krebsbach[1,2], Mikel Rincón-Iglesias[1,2,3], Manuel Pietsch[1,2], Carmen Henel[1,2], Senentxu Lanceros-Mendez[3,4], Jun Wei Phua[5], Marianna Ambrico[6], Gerardo Hernandez-Sosa[1,2,7]**

[1]Light Technology Institute, Karlsruhe Institute of Technology, Engesserstr. 13, 76131 Karlsruhe, Germany.
[2]InnovationLab, Speyerer Straße 4, 69115 Heidelberg, Germany.
[3]BCMaterials, Basque Center for Materials, Bldg. Martina Casiano, UPV/EHU Science Park Barrio Sarriena s/n, 48940 Leioa, Spain.
[4]IKERBASQUE, Basque Foundation for Science Plaza Euskadi 5, Bilbao 48009, Spain.
[5]Insectta Pte Ltd., 77 Ayer Rajah Crescent, Singapore 139954, Singapore.
[6]Institute for Plasma Science and Technology (CNR- ISTP), National Research Council of Italy, Via Amendola 122/D, 70126 Bari, Italy.
[7]Institute of Microstructure Technology, Karlsruhe Institute of Technology, 76344 Eggenstein-Leopoldshafen, Germany.

*Email: gerardo.sosa@kit.edu

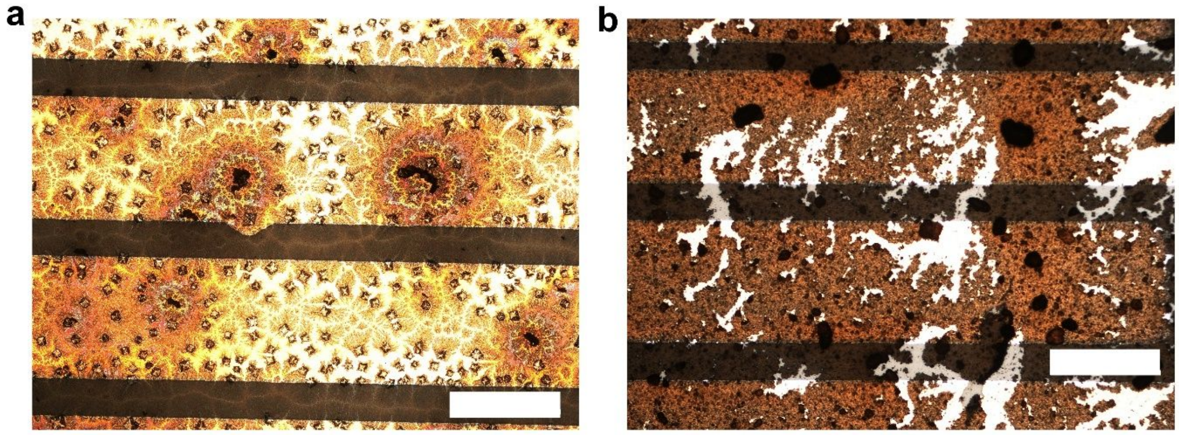


**Figure S1.** Examples of spin coating of BSF-Melanin composites with other salts than ChCl resulting in a less homogeneous film formation: **a** NaCl and **b** $CaCl_2$.

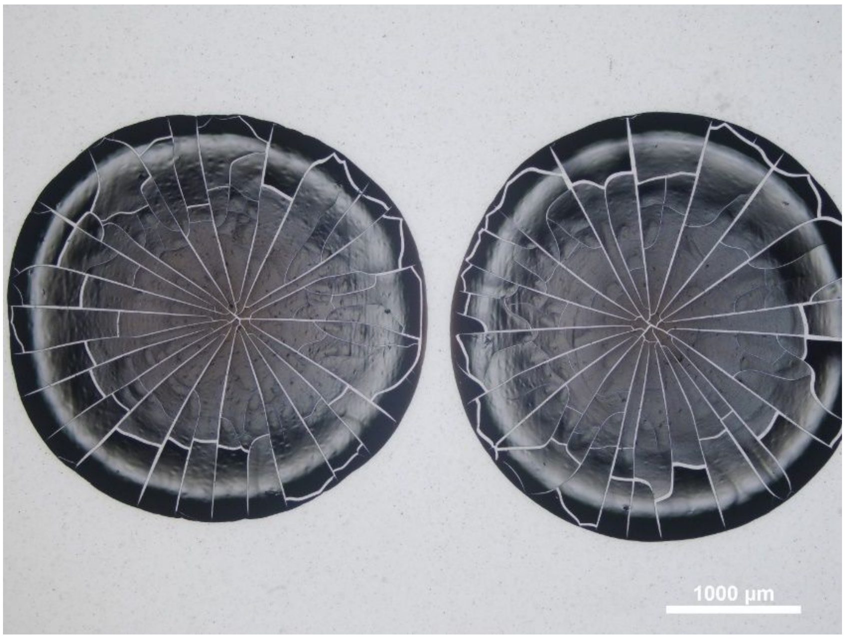


**Figure S2.** Drop-cast BSF-Melanin can result in cracked layers upon drying for high concentrations.

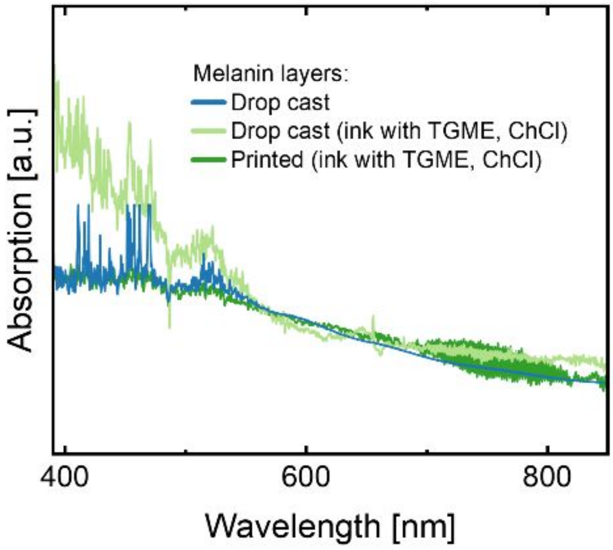


**Figure S3.** UV-Vis spectra of BSF-Melanin layers with and without additives. The spectra were measured using a deuterium halogen light source and fiber spectrometer (AvaLight-DH-S-BAL, AvaSpec-ULS3648, Avantes).

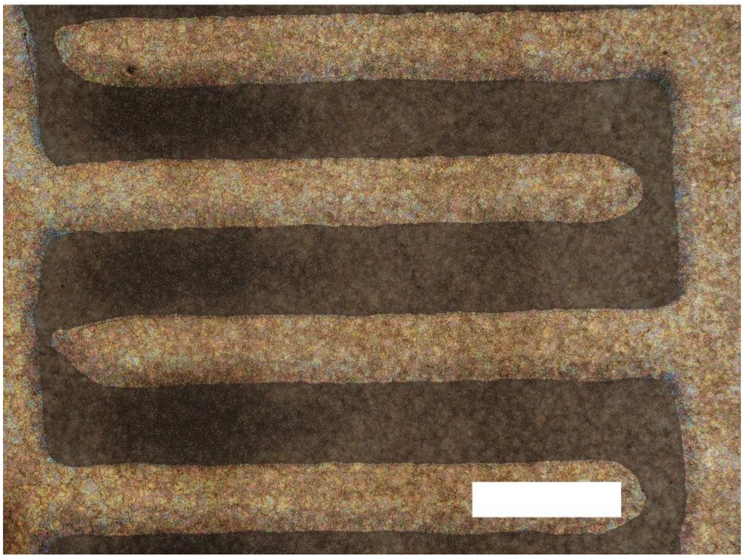

**Figure S4.** Inkjet-printed BSF-Melanin layer from BSF-Melanin-water-TGME dispersion. The scale bar is 500 μm.

**Note S1: EIS data elaboration via broadband dielectric spectroscopy (BDS) approach**

The EIS data was analyzed by broadband dielectric spectroscopy (BDS) through a comparative study of the complex dielectric permittivity $\varepsilon^*$ and the complex AC conductivity $\sigma^*$. The dielectric permittivity $\varepsilon^*$ is expressed by the real $\varepsilon'$ and imaginary part $\varepsilon''$ of the permittivity as in the following equation:

$$\varepsilon^* = \varepsilon' - i\varepsilon'' , \quad (S1)$$

where $\varepsilon'$ and $\varepsilon''$ represent the storage and loss components, respectively, calculated via the experimental $Z'$ and $Z''$ vs. frequency dispersions following the classical expressions:[1]

$$\varepsilon' = \frac{-Z''}{2\pi f\,|Z|^2 C_0} , \quad (S2)$$

$$\varepsilon'' = \frac{-Z'}{2\pi f\,|Z|^2 C_0} . \quad (S3)$$

with the geometrical vacuum cell capacitance $C_0$ and the squared absolute $Z$. In the present case, we estimated these values taking into consideration the ones measured for an empty IDE electrode ($C_0$ = 100 pF).

The complex AC conductivity $\sigma^*$ is determined via the $\varepsilon^*(\omega)$ using the expression:[1]

$$\sigma^*(\omega) = \sigma'(\omega) + i\sigma''(\omega) = i\omega\varepsilon_0\varepsilon^*(\omega) = i\omega\varepsilon_0\big(\varepsilon' - i\varepsilon''(\omega)\big)\ . \qquad \text{(S4)}$$

Here, the Trukhan model is adopted,[2] although it may result in the overestimation of the free ionic charge diffusion coefficient $D$ and their density.[3] However, this approach still stays valid for a prompt evaluation of the response of ionic conducting systems as demonstrated by its application in a variety of ionic conducting polymeric materials and ionic liquids.[4,5]

Following Trukhan, the diffusion D is given by:

$$D = \frac{\omega_{max}\, L^2}{32\, N_{pair}\, (\tan\,(\delta_{\max}\,))^3}\ . \qquad \text{(S5)}$$

With $L$ representing the distance between electrodes and $\omega_{\max}$ the angular frequency of the maximum value of tan($\delta$). As it can be noticed, we slightly modified the original expression to account for the IDE configuration of the device; more specifically, $D$ and $L$ referred to a single pair of fingers ($N_{\text{pair}}$ = 7), and the distance between each pair of fingers (~ 350 μm).

The ionic charge concentration $n_{\text{ion}}$ was derived from:

$$n_{\text{ion}} = \frac{\sigma'_{\text{DC}}}{D} \cdot \frac{k_{\text{B}}T}{e^2}\ , \qquad \text{(S6)}$$

where $\sigma'_{\text{DC}}$ is the real part of the AC conductivity at the plateau and taken at the radial frequency $\omega_{\max}$ of the maximum of the loss factor, $k_{\text{B}}$ the Boltzmann constant, $T$ the temperature, and $e$ the elementary charge. The ionic charge mobility $\mu$ is calculated as:

$$\mu = \frac{\sigma'_{\text{DC}}}{e\, n_{\text{ion}}}\ . \qquad \text{(S7)}$$

## References:


(1) Barsoukov, E.; Macdonald, J. R. *Impedance Spectroscopy: Theory, Experiment, and Applications*; John Wiley & Sons, 2018.

(2) Trukhan, E. Dispersion of the Dielectric Constant of Heterogeneous Systems. *Soviet Physics - Solid State* **1963**, *4*, 2560.

(3) Sørensen, T. S.; Compañ, V. Complex Permittivity of a Conducting, Dielectric Layer Containing Arbitrary Binary Nernst–Planck Electrolytes with Applications to Polymer Films and Cellulose Acetate Membranes. *J. Chem. Soc., Faraday Trans.* **1995**, *91* (23), 4235–4250. https://doi.org/10.1039/FT9959104235.
(4) Pal, P.; Ghosh, A. Broadband Dielectric Spectroscopy of BMPTFSI Ionic Liquid Doped Solid-State Polymer Electrolytes: Coupled Ion Transport and Dielectric Relaxation Mechanism. *Journal of Applied Physics* **2020**, *128* (8), 084104. https://doi.org/10.1063/5.0022027.
(5) Ambrico, M.; Mostert, A. B.; Ambrico, P. F.; Phua, J.; Mattiello, S.; Gunnella, R. Exploring Ion Mobility Mechanisms in Poly Indolequinone Polymers: A Case Study on Black Soldier Fly Melanin. *J. Phys. D: Appl. Phys.* **2024**, *57* (26), 265303. https://doi.org/10.1088/1361-6463/ad3765.

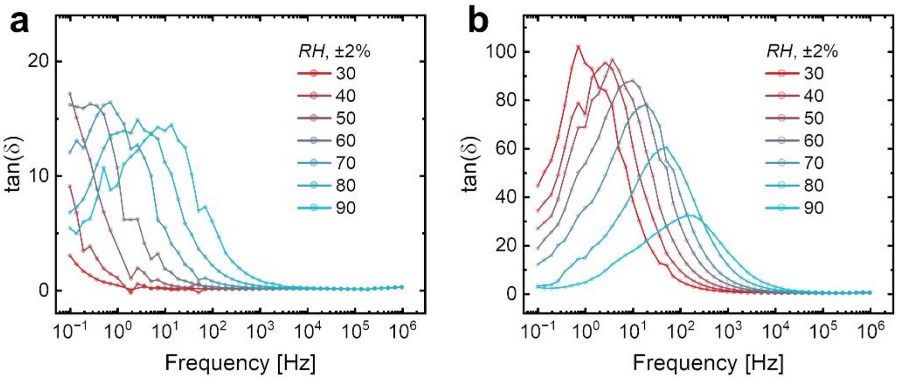


**Figure S5.** Loss factor extracted for **a** BSF-Melanin sensors and **b** BSF-Melanin-ChCl sensors as a function of frequency and RH.

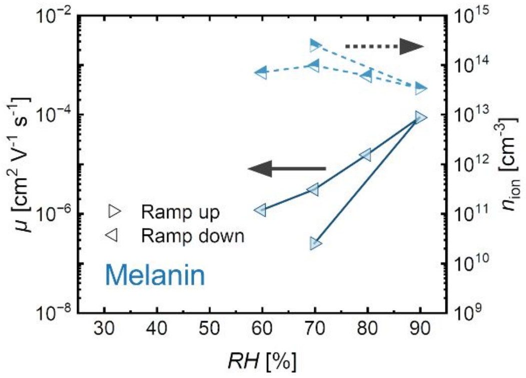


**Figure S6.** Mobility $\mu$ and $n_{\text{ion}}$ for pristine BSF-Melanin humidity sensors extracted from EIS data. No values below 60 % RH could be accurately determined for the pristine BSF-Melanin sensor because the maximum of the loss factor, which was needed for the calculation, lay at lower frequencies (< 0.1 Hz) than the measured data (Equation S6 and S7, Figure S4).

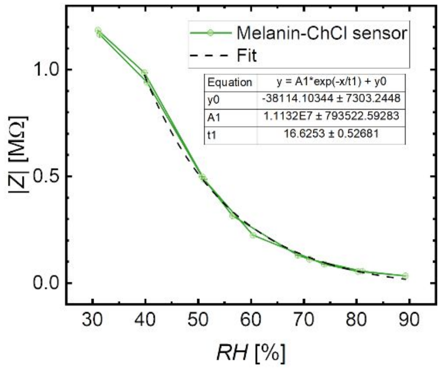


**Figure S7.** Calibration curve for the sensor used in the dynamic setup. Data points at 30 % RH are excluded for a better fit as the detection limit is reached for 30 % RH at 1 kHz. Variable x in the fitting equation equals RH.

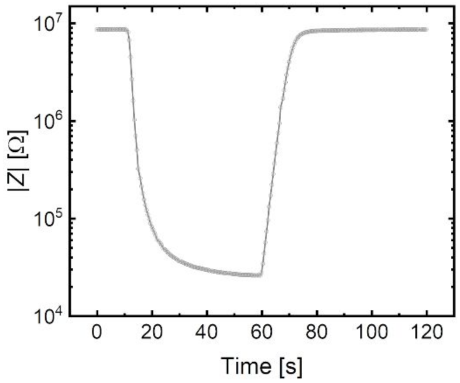


**Figure S8.** Dynamic measurement of the reference sensor with a longer timespan to calculate $t_{90}$.

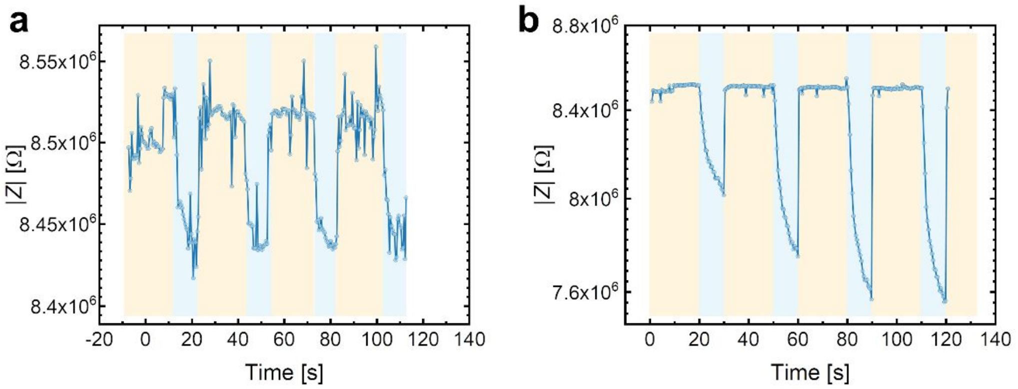


**Figure S9.** Two (**a+b**) representative dynamic measurements of pure BSF-Melanin sensors. Orange background for dry, blue for wet $N_2$ flow.

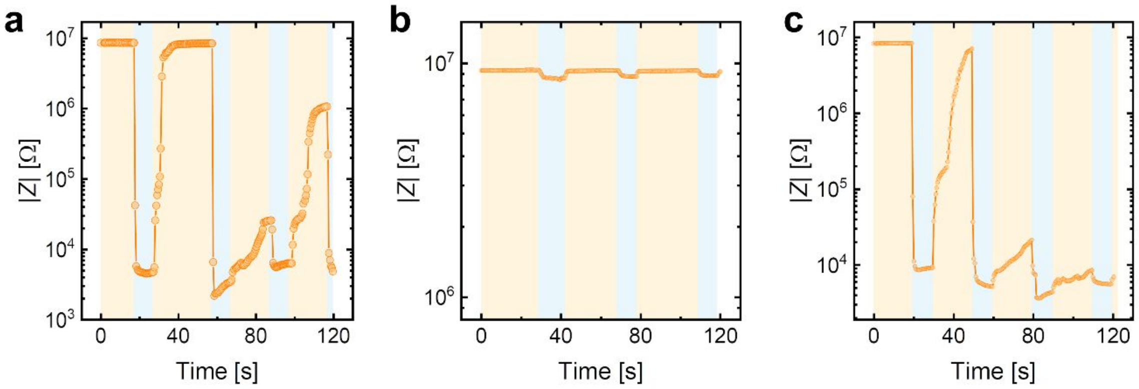


**Figure S10.** Three (**a+b+c**) exemplary dynamic measurements of pure ChCl sensors. Orange background for dry, blue for wet $N_2$ flow.

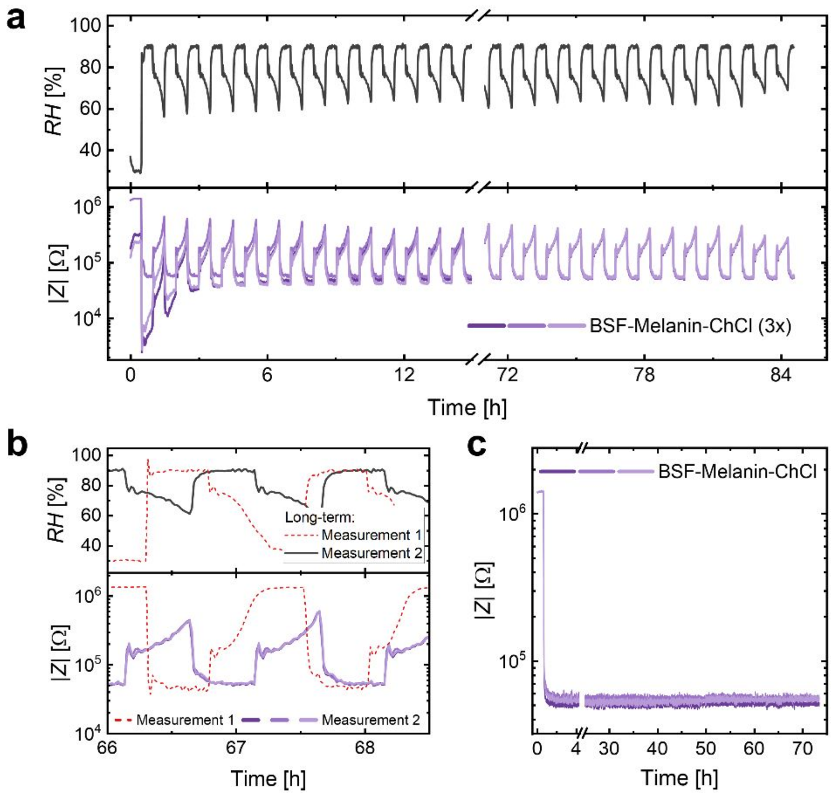


**Figure S11. a** Long-term measurement of three BSF-Melanin-ChCl sensors at higher average humidity compared to the data shown in the manuscript. The climate chamber was set to increase humidity to 90 % for 30 min and to decrease down to 30 % for another 30 min (yet, 30 % were not reached). The top panel shows the data of the climate chamber. The bottom

panel shows the BSF-Melanin-ChCl sensors. **b** Last hours of measurement 1 (manuscript) and measurement 2 (from Figure S11a) in comparison. **c** Three sensors (as Figure S11a) recorded at constant 90 % RH over three additional days.

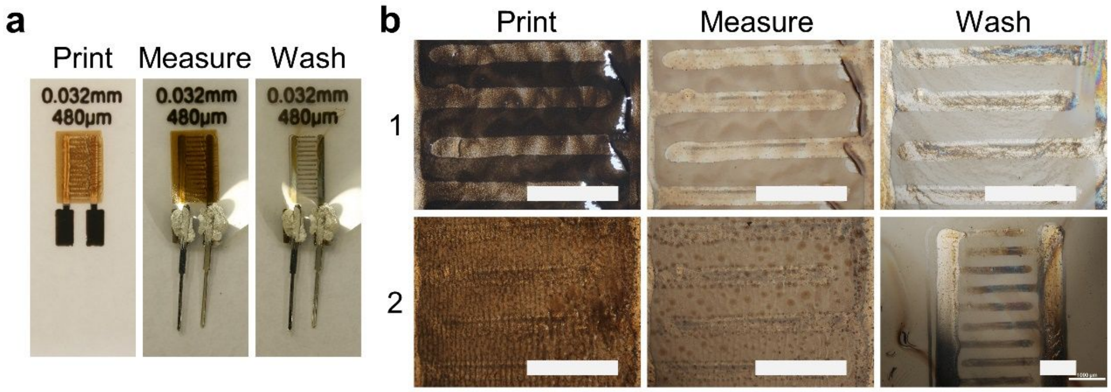


**Figure S12. a** Photographs of BSF-Melanin-ChCl sensor after printing, measuring in the climate chamber, and washing in a water bath. **b** Micrographs of the same sensor during two cycles of printing, measuring, and washing. The scale bar in all micrographs is 1 mm.

## Note S2: Differences for BSF-Melanin-ChCl Films on Cellulose Diacetate/Au IDEs

The films for sensors on the biodegradable substrate were fabricated at the beginning of the study still using the Fujifilm legacy DMC 16110 printheads. The nozzle volume on these was larger than for the printheads used for the other sensors in this work so the requirements for the ink were different and the deposition from a pure water-based ink was possible. Hence, these sensors were not deposited from ink including TGME. Nevertheless, relative concentrations of BSF-Melanin and ChCl to each other and to water were held constant. The change to the new DMC Samba led to a necessary adaptation of the ink. The Au IDEs were printed with 1 pl legacy cartridges at 1500 dpi. Due to differences in nozzle spacing and volume, the printing resolution had to be adapted for each of the mentioned printheads also changing final film thicknesses.

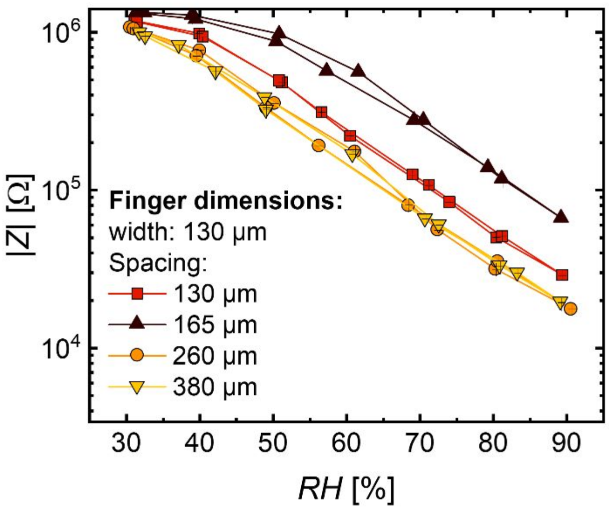


**Figure S13.** Variation of finger spacing of the IDEs and their according performance. Tests were done with Fujifilm DMC legacy printheads for silver printing (optimization by form and conductivity). The final IDE in manuscript differs slightly due to a different printhead (Fujifilm DMC Samba).
